# Cervicocephalic kinaesthesia reveals novel subgroups of motor control impairments in patients with neck pain

**DOI:** 10.1038/s41598-024-57326-1

**Published:** 2024-04-10

**Authors:** Ziva Majcen Rosker, Jernej Rosker

**Affiliations:** 1 Faculty of Sport, University of Ljubljana, Ljubljana, Slovenia; 2 Faculty of Health Sciences, University of Primorska, Koper, Slovenia

**Keywords:** Neuroscience, Diseases, Health care, Signs and symptoms

## Abstract

Cervical-spine sensorimotor control is associated with chronicity and recurrence of neck pain (NP). Tests used to measure sensorimotor impairments lack consistency in studied parameters. Interpretation is often based on either a handful or numerous parameters, without considering their possible interrelation. Different aspects of motor-control could be studied with different parameters, but this has not yet been addressed. The aim of this study was to determine if different parameters of cervical position (JPE) and movement (Butterfly) sense tests represent distinct components of motor-control strategies in patients with chronic NP. Principal component analysis performed on 135 patients revealed three direction-specific (repositioning from flexion, extension or rotations) and one parameter-specific (variability of repositioning) component for JPE, two difficulty-specific (easy or medium and difficult trajectory) and one movement-specific (undershooting a target) component for Butterfly test. Here we report that these components could be related to central (neck repositioning and control of cervical movement) and peripheral sensorimotor adaptations (variability of repositioning) present in NP. New technologies allow extraction of greater number of parameters of which hand-picking could lead to information loss. This study adds towards better identification of diverse groups of parameters offering potentially clinically relevant information and improved functional diagnostics for patients with NP.

## Introduction

Motor control characteristics of the cervical spine have been extensively studied in patients with neck pain disorders^1–3^. To date articles report on alterations in different subsets of motor control tasks of which cervicocephalic kinaesthetic acuity plays a crucial role in the development of chronicity and recurrence of neck pain^4,5^. Kinaesthetic sensibility is a complex functional ability consisting of different aspects such as position and movement sense^6^ both integral components of motor control^7^. Studies focusing on identifying kinaesthetic impairments of the cervical spine cover different aspects of motor control but lack aspect specific interpretation.

Recent systematic literature review reports diverse results in various aspects of cervical motor control in patients with neck pain depicted by changes in many different parameters^8^. While position sense tests commonly apply parameters of variable, constant and absolute error^9,10^ movement sense tests apply more diverse parameters such as smoothness of movement^11^, accuracy of head and neck movement^2,12,13^ directional accuracy^14^, mean and peak velocity of head movement^12,13,15^ time to peak velocity^12,16^ and others, although all these are not consistently applied throughout the literature. Some studies apply limited amount of parameters^11,13,17^, while other studies apply numerous parameters simultaneously^18–20^ which raises the concern of addressing different characteristics of kinaesthetic sensibility, however to our knowledge this has not yet been addressed.

In the recent study by de Zoete et al^21^. an attempt was made to better understand if various cervical spine motor control tests measure similar or different skills in patients with neck pain. Although in their study variety of motor control tests were analyzed, head and neck movement sense but not position sense tests presented a separate entity. Movement and position sense tests in their study were analyzed using only one parameter (mean amplitude accuracy and mean error respectively) decreasing sensitivity of these tests by possibly excluding other integral parts of movement and position sense. Similar inconclusive results regarding alterations in cervical joint position sense were presented in previous meta-analysis and systematic reviews^10,22,23^. Based on the above, more in-depth understanding of individual parameters describing specific subsets of kinaesthetic impairments should be studied in a heterogenous group of patients with neck pain.

Aforementioned rationales are additionally supported by more in-depth understanding of the underlying mechanisms of the position and movement sense which are vital for kinaesthetic acuity. Commonly, cervical position sense is thought to be largely dependent on cervical sensory input from different mechanoreceptors and their integration at higher levels of central nervous system^24^. However, as suggested by the equilibrium point hypothesis, basic understanding of mechanisms governing position sense have been undermined^25^. Namely, centrally generated perceptual frame of the head and neck posture (reference position) is suggested to be the origin of position sense while peripheral information derived from cervical mechanoreceptors signal possible mismatch between the actual and reference position^7,25^.

Diverse alterations in the underlying mechanisms of the joint position sense have additionally been suggested by nonhomogeneous changes in various position sense parameters studied in patients with neck pain^9^. Therefore, it would be of importance to analyze whether different parameters describing position sense could indicate different aspects in joint position sense disturbances in patients with neck pain.

On the contrary to above described characteristics of position sense, movement sense tests require high accuracy of head and neck movements, which are commonly performed with increased neck stiffness in patients with neck pain^1,8,26^. Increased stiffness of the neck is thought to be accompanied by increased neck muscle coactivation^27,28^ which has been proposed to lead to decreased accuracy of movement^7,29^ and can be even more pronounced at higher movement velocities^30^. Therefore, it would be of importance to better understand whether accuracy of head and neck movements at different predetermined velocities and increasing difficultness of reference movement trajectory is representative of diverse subsets of motor control.

The aim of this study is to analyze whether various parameters of head and neck movement sense test and position sense test present with similar or different components of motor control strategies and whether the predefined movement difficultness of movement sense test provides additional insights into motor control deficits in patients with neck pain.

## Results

### Patient demographics

One hundred and thirty-five patients with idiopathic neck pain participated in this study with their demographic data presented in Table 1. No statistical differences in VAS level were observed between the three subgroups of patients with idiopathic neck pain.

**Table 1:** demographic data of the enrolled patients.

Subgroup/group	Number	VAS score (average ± standard deviation)	Number of females	Number of males
Orthopedic outpatient clinics	45	4.51 ± 1.50	21	24
Physiotherapy clinics	45	4.77 ± 1.32	23	22
Ergonomic environment	45	4.40 ± 1.55	24	21
Together	135	4.56 ± 1.46	68	67

VAS—score on visual analogue scale.

### Principle component analysis of the position sense test

**Figure 1:**
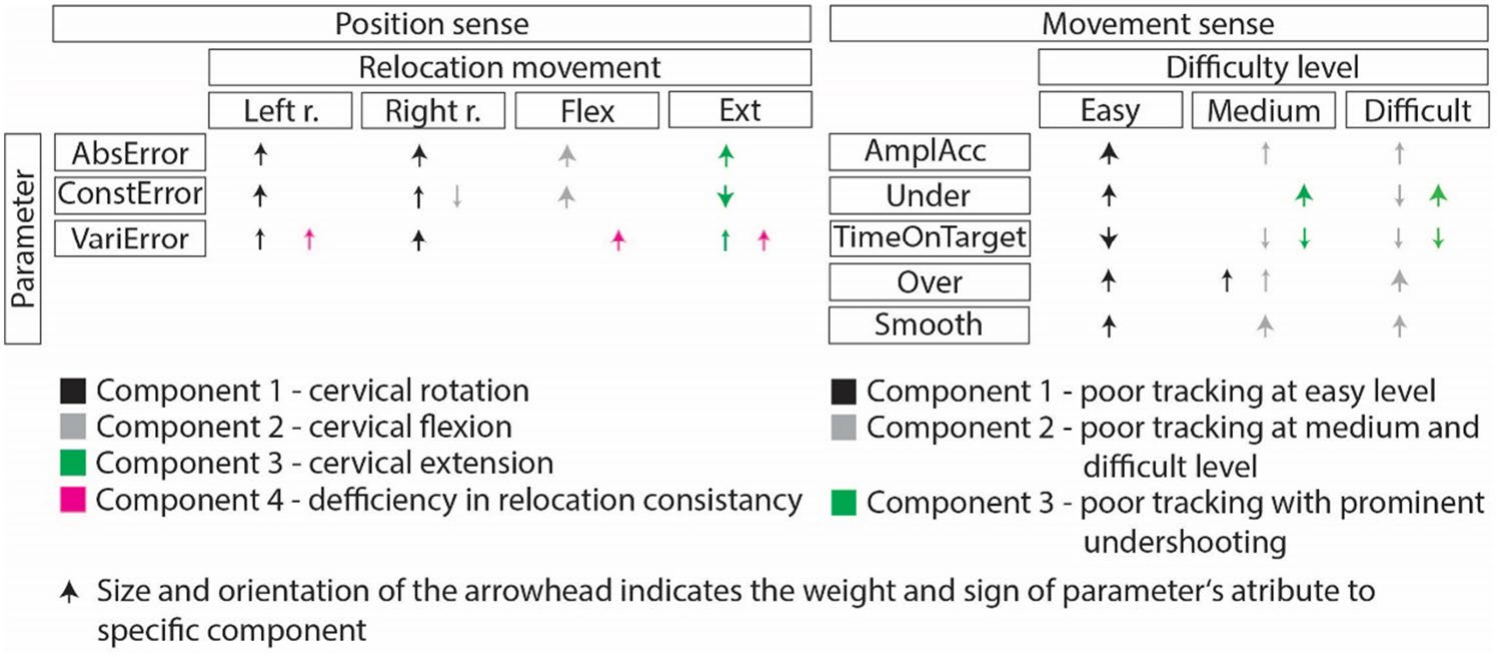
presentation of principle components for the cervical position sense test and the Butterfly test performed simultaneously AbsError—absolute error; ConstError—constant error; VariError—variable error; _left—relocation from left rotation; _right—relocation from right rotation; _flexion—relocation from flexion; _extension—relocation from extension; AmplAcc—amplitude accuracy; TimeOnTarget—time on target; Under—undershoot; Over—overshoot; Smooth—smoothness of movement.

Results of principle component analysis of the position sense test are presented in Table 2 and Fig. 1. Four components were identified. The first component consisted of all errors for left and right head rotation with positive weights, suggesting that this component consisted of decreased head relocation accuracy for both rotations. The second component consisted of positive weights for absolute and constant error for relocation from extension and negative weight for constant error for right rotation. The third component presented with positive weights for absolute error and variable error when relocating from flexion. In this component a large negative weight for constant error from extension was observed. The fourth component included positive weights for variable error from flexion, extension and left rotation, depicting the altered ability to relocate patients head and neck primarily in the sagittal plane.

**Table 2:** principal component analysis for the cervical position sense test.

	Principal components (weight)			
	1	2	3	4
AbsError_left	0.693			
ConstError_left	0.739			
VariError_left	0.473			0.553
AbsError_right	0.841			
ConstError_right	0.526	-0.425		
VariError_right	0.800			
AbsError_flexion		0.958		
ConstError_flexion		0.920		
VariError_flexion				0.820
AbsError_extesnion			0.823	
ConstError_extension			-0.905	
VariError_extension			0.429	0.661
Eigenvalue	3.775	2.305	1.402	1.119
% Variance Explained	31.46	19.206	11.682	9.322

*AbsError*—*absolute error; ConstError*—*constant error; VariError*—*variable error; _left*—*relocation from left rotation; _right*—*relocation from right rotation; _flexion*—*relocation from flexion; _extension*—*relocation from extension; % Variance Explained*—*percentage of total variance explained by an individual principle component.*

### Principle component analysis of the Butterfly test

The results of principal component analysis when parameters of the Butterfly test were included are presented in Table 3. In this analysis, three components with eigenvalues above 1 were identified. First component consisted primarily of all parameters describing easy difficulty level of the Butterfly test. More specifically, increased amplitude accuracy, smoothness of movement, undershoot and overshoot were observed in this component accompanied by decreased time on target. In the second component, similar trend was observed as in component one, only that the second component was related to medium and difficult level of the Butterfly test. In the third—last component, high undershoot and low time on target at medium and difficult level of the Butterfly test were observed. In the results it is evident that increase in amplitude accuracy, smoothness of movement, undershoot, overshoot and decreased time on target represent altered represent important alterations in patient sample. In addition, comparison between the first and second component suggests that difficulty level of the Butterfly test also represents a separate factor. The last component suggests that increased undershoot accompanied by decreased time on target at medium and difficult levels represents specific movement control deficit.

**Table 3:** principal component analysis for the Butterfly test.

	Component		
	1	2	3
AmplAcc_easy	1.047	–	–
AmplAcc_med	–	0.437	–
AmplAcc_diff	–	0.514	–
TimeOnTarget_easy	− 0.952	–	–
TimeOnTarget _med	–	− 0.439	− 0.583
TimeOnTarget _diff	–	− 0.455	− 0.625
Under_easy	0.790	–	–
Under_med	–	–	0.932
Under_diff	–	− 0.521	0.957
Over_easy	0.792		–
Over_med	0.567	0.406	–
Over_diff	–	0.944	–
Smooth_easy	0.757		–
Smooth_med	–	0.914	–
Smooth_diff	–	0.767	–
Eigenvalue	8.453	2.296	1.355
% Variance Explained	56.354	15.305	9.034

*AmplAcc*—*amplitude accuracy; TimeOnTarget*—*time on target; Under*—*undershoot; Over*—*overshoot; Smooth*—*smoothness of move0ment; _easy*—*easy level of the Butterfly test; _med*—*medium level of the Butterfly test; _diff*—*difficult level of the Butterfly test; % Variance Explained*—*percentage of total variance explained by an individual principal component.*

### Principle component analysis of the position sense test and butterfly test performed simultaneously

In Table 4 results of principal component analysis when considering the Butterfly test and cervical position sense test together are presented. No major changes were observed as compared to previous analysis. The main difference observed was that head and neck relocation from extension and flexion combined in one component (component 5). In addition, according to the size of eigenvalues, the most prominent component explaining the largest proportion of variance was the one indicating accuracy of head and neck movement at the easy level of the Butterfly test. This was followed by the head and neck relocation accuracy from rotation. Other components presented with similar size of eigenvalues.

**Table 4:** principal component analysis for the cervical position sense test and the Butterfly test performed simultaneously.

	Component					
	1	2	3	4	5	6
AbsError_left	–	0.947	–	–	–	–
ConstError_left	–	0.831	–	–	–	–
VariError_left	–	0.921	–	–	–	–
AbsError_right	–	0.883	–	–	–	–
ConstError_right	–	0.769	–	–	–	–
VariError_right	–	0.895	–	–	–	–
AabsError_flexion	–	–	–	–	0.791	–
ConctError_flexion	–	–	–	–	0.889	–
VariError_flexion	–	–	–	–		0.789
AabsError_extension	–	–	− 0.462	–	0.675	–
ConstError_extension	–	–	–	–	− 0.735	–
VariError_extension	–	–	–	–	–	0.746
AmplAcc_easy	1.002	–	–	–	–	–
AmplAcc_med	0.403	–	0.412	–	–	–
AmplAcc_diff		–	0.435	–	–	–
TimeOnTarget_easy	− 0.915	–	–	–	–	–
TimeOnTarget_med		–	–	− 0.607	–	–
TimeOnTarget_diff		–	− 0.421	− 0.653	–	–
Under_easy	0.728	–	–		–	–
Under_med		–	–	0.940	–	–
Under_diff		–	–	0.926	–	–
Over_easy	0.805	–	–		–	–
Over_med	0.590	–	0.407		–	–
Over_diff		–	0.752		–	–
Smooth_easy	0.844	–	–	− 0.416	–	–
Smooth_med		–	0.866		–	–
Smooth_diff		–	0.713		–	–
Eigenvalue	8.631	5.281	2.679	2.29	1.587	1.317
% Variance Explained	31.966	19.558	9.924	8.483	5.878	4.879

*AbsError*—*absolute error; ConstError*—*constant error; VariError*—*variable error; _left*—*relocation from left rotation; _right*—*relocation from right rotation; _flexion*—*relocation from flexion; _extension*—*relocation from extension; AmplAcc*—*amplitude accuracy; TimeOnTarget*—*time on target; Under*—*undershoot; Over*—*overshoot; Smooth*—*smoothness of movement; _easy*—*easy level of the Butterfly test; _med*—*medium level of the Butterfly test; _diff*—*difficult level of the Butterfly test; % Variance Explained*—*percentage of total variance explained by an individual principle component.*

### Component correlation analysis

Correlation matrix for components of the position sense and movement sense tests are presented in Table 5. For the components of the position sense, small correlations were observed between the second and third component. For the movement sense test small correlations were observed between the first and the third component as well as between the second and the third component. However, medium correlations were observed between the second and the first component. Correlation matrix for components of both cervical position sense and the Butterfly test analyzed simultaneously presented with small correlations between first and third component as well as between first and fourth component.

**Table 5:** component correlation matrix.

		Component					
	Component	1	2	3	4	5	6
Position sense	1	1.00	.121	.024	.267	–	–
	2	.121	1.00	.432	.175	–	–
	3	.024	.432	1.00	.157	–	–
	4	.267	.175	.157	1.00	–	–
Butterfly test	1	1.00	.626	.467		–	–
	2	.626	1.00	.333		–	–
	3	.467	.333	1.00		–	–
Position sense and Butterfly test	1	1.00	− .083	.498	.493	.073	.036
	2	− .083	1.00	.074	− .099	.196	.151
	3	.498	.074	1.00	.24	.109	.152
	4	.493	− .099	.240	1.00	− .044	− .055
	5	.073	.196	.109	− .044	1.00	.147
	6	.036	.151	.152	− .055	.147	1.00

## Discussion

In the present study subgroups of different parameters from the cervical position sense test and the Butterfly test (i.e. movement sense test) were identified. Additionally, the effect of difficulty level during the Butterfly test on parameter’s subgrouping were studied. Based on the results from our study, parameters of both; cervical position sense test and the Butterfly test demonstrated separate components with no mixing of parameters from the two tests. These results suggest that studied parameters represent separate motor control entities. When studied separately, cervical position sense test presented with four components of parameters while the Butterfly test presented with three components. Moreover, when merged in the analysis all together no important changes in the identified components were observed.

### Cervical position sense

Cervical position sense test is commonly used in research and clinical practice^31,32^ but the interpretation of parameters is usually scarce. Cervical position sense test is primarily interpreted based on the movement direction specific deficits (i.e. flexion, extension, rotation) but less attention is placed on identifying alterations based on individual parameters, such as absolute, constant and variable error. Based on the results from our study, direction specific alterations as well as individual parameters contribute towards identifying underlying impairments in patients with neck pain. Results of the principal component analysis presented with four components in our study. First component described impairments in both cervical rotations, while second and third components were indicative of deficits in relocating patient’s cervical spine from flexion and extension respectively. The last component presented with deficits in consistency (variable error) between repeated trials when relocating from flexion and extension. Components observed in the cervical position sense test suggest that repositioning error from flexion, extension and both rotations represent separate entities which is also confirmed by small correlations between individual components. These results are somewhat expected as previous studies suggested that structure, location and type specific impairments can be found in those with cervical spine disorders^4,24,33,34^. The latter could present with motor control deficits when relocating from certain directions.

In addition to direction specific deficits, our results show that absolute and constant error represent same characteristics of the cervical position sense test, while variable error represents a separate entity. This seems logical, since absolute and constant error express repositioning error relative to the reference position, while variable error measures consistency between consecutive repositions. Moreover, the different nature of these three repositioning parameters can be partially explained by the equilibrium point hypothesis. According to this hypothesis, perception of position is based on centrally produced salient feature determining the reference joint position^7^ and its difference from the actual joint position^25^. The latter is determined by interaction between joint stiffness characteristics and external forces acting on a joint^7,25^. Joint stiffness is suggested to be altered in patients with neck pain, possibly due to increased cervical muscle coactivation^28,35^. The latter could be a consequence of decreased cervical spine stability and decreased precision of reference position control^29^. Therefore, it is tempting to relate absolute and constant error to altered control of reference joint position (i.e., central adaptations). Variable error on the other hand could be related to inconsistent sensory feedback from the periphery (e.g., mechanoreceptors from cervical muscles, intervertebral discs, ligaments etc.), that has been observed in patients with neck pain^24,33^. This could lead to variability in actual position perception on a movement to movement basis^25^ and therefore larger dispersion between consecutive repositions.

### Cervical movement sense

Results of principle component analysis performed on the Butterfly test’s parameters presented with three components. These differed primarily on the difficulty level of the test with easiest level presenting first component, while medium and difficult levels presented second component. Both components were characterized by decreased time spent on target, increased time of overshooting when tracking a target, increased jerkiness of movement and increased distance between the target and head position. The third component indicated the presence of movement specific deficits that consisted of undershooting the target accompanied by less time spent on the target at the medium and difficult levels.

Results from principal component analysis where easiest level of the Butterfly test presented a separate entity as compared to medium and difficult levels can be supported by findings from studies focusing on synergistic muscle activity and their relation to speed-accuracy trade-off^7,29,36^. Muscle synergies consist of muscle grouping with a certain spatial and temporal goal of movement (e.g. direction of discrete movements). In the Butterfly test unpredictable multidirectional head movements (i.e. velocity and direction changes) demand online control of tracking accuracy and fine-tuned shifts between direction specific muscle synergies^7,30^. Additionally, muscle synergies need to be adapted to temporal constraints^36,37^ which is distinctive for the Butterfly test.

Furthermore, increased difficulty of a movement task can induce shifts from direction specific to more generalized muscle synergies, resulting in increased stiffness of the body^30,37^. More pronounced shifts towards generalized muscle synergies at easier movements could be expected in patients with neck pain. This notion is supported by studies reporting increased muscle coactivation during different unidirectional tasks^28,38^ and decreased complexity of cervical muscle representation in motor cortex, implying lower versatility of muscle control for different contexts^27^. These could lead to less direction specific muscle synergies. It can be speculated that easy level of the Butterfly test could enable more efficient coordination between direction specific muscle synergies. On the contrary, medium and difficult levels of The Butterfly test likely lead to increase in muscle coactivation and more generalized muscle activity. The Butterfly test could therefore indicate how well can direction specific muscle synergies be controlled at different difficulty levels.

In addition, based on the above it could be speculated, that increased muscle coactivation in patients with neck pain enable more abundant proprioceptive feedback via increased number of simultaneously active neck muscles. This could present a strategy to at least partially counteract above mentioned side or direction specific proprioceptive deficits in patients with neck pain^4,24^ positively contributing to accuracy of head and neck movements.

In addition to alterations in proprioceptive information discussed above, sensory mismatch can be present in patients with neck pain as a result of functional adaptations in visual and vestibular systems^39,40^. Butterfly test requires tracking an unpredictably moving target of constantly changing velocity, acceleration and direction of movement. According to Wibble et al^41^. acceleration of visual stimuli affects interplay between different sensory sources, increasing dependance on online visual feedback in order to perceive movement and head position during the Butterfly test. Cervical spine pathology related oculomotor deficits have been reported in patients with neck pain^42–45^ and could negatively affect the accuracy of visual feedback. More specifically, patients with neck pain have been shown to have decreased accuracy of smooth pursuit eye movements accompanied by increased amount of fast saccadic eye movements, which can alter timely perception of target movement^46,47^. Such deficits could lead to increased application of lagging behind the target (undershooting) as has also been suggested by the fourth component of the combined principal component analysis.

### Clinical implications

Our findings have important implications for clinical practice due to new knowledge in identifying sensorimotor deficits in patients with neck pain. In recent years wearable sensor technologies were introduced in research and clinical practice upgrading previous analogous approaches. New technologies have allowed extraction of greater number of parameters which was believed to positively influence sensitivity and to better identify different aspects of motor control in patients with neck pain^20,48^. Unfortunately, it was unclear whether any of these parameters present similar features of cervical sensorimotor control which was addressed in our study. This is very important when collecting multiple parameters^14,20^ but reducing it to only few^21^ since hand picking of parameters could potentially lead to information loss.

Important limitations of our study were that only one cervical position sense test and one cervical movement sense test were used despite literature reporting use of other additional tests^8,20,21^. Although parameters among tests may present some similar features, more tests should be included in the future to better understand different characteristics of sensorimotor control and possible differences between various tests.

Since sensorimotor control is commonly investigated in patients with neck pain disorders but less in other pathologies, important limitation of our study was that only patients with chronic neck pain were enrolled in the study. Future studies should also consider performing principal component analysis on a variety of cervical sensorimotor control tests in different groups of asymptomatic individuals as well as other patients with cervical spine impairments and those with vestibular and visual disorders.

Based on our findings, specific subcomponents of different sensorimotor control tests were identified that need further clarification in order to enable design of impairment specific rehabilitation protocols. This study importantly adds towards better identification and understanding on how to apply and interpret different parameters from cervical position and movement sense tests in research and clinical practice when treating patients with chronic neck pain.

## Methods

### Participants

Sample size applied in this study was determined using the rule of 5 participants per parameter, which resulted in measuring 135 patients with neck pain^49^. As patients with idiopathic neck pain present a heterogenous group, three different recruitment approaches were considered in this study. Patients who reported neck pain for longer than 3 months (chronic) were recruited from orthopedic outpatient clinics, physiotherapy clinics (without referral) and ergonomic environment (office workers from three different companies). Equal number of patients was included from all recruitment approaches. Age range for the enrollment in the study was 18–65 years of age. To be considered for the study, patients had to report pain intensity of at least 3 out of 10 on visual analogue scale. In addition, patients had to be free from head and shoulder injuries within the last two years and had to be off medication for at least 30 h before the study. Informed consent was obtained from all subjects prior to enrolment. The study was performed in accordance with declaration of Helsinki and its later amendments and received approval by the National Medical Ethics Committee of the Republic of Slovenia (number: 0120–47/2020/6).

### Measurements and procedures

**Figure 2:**
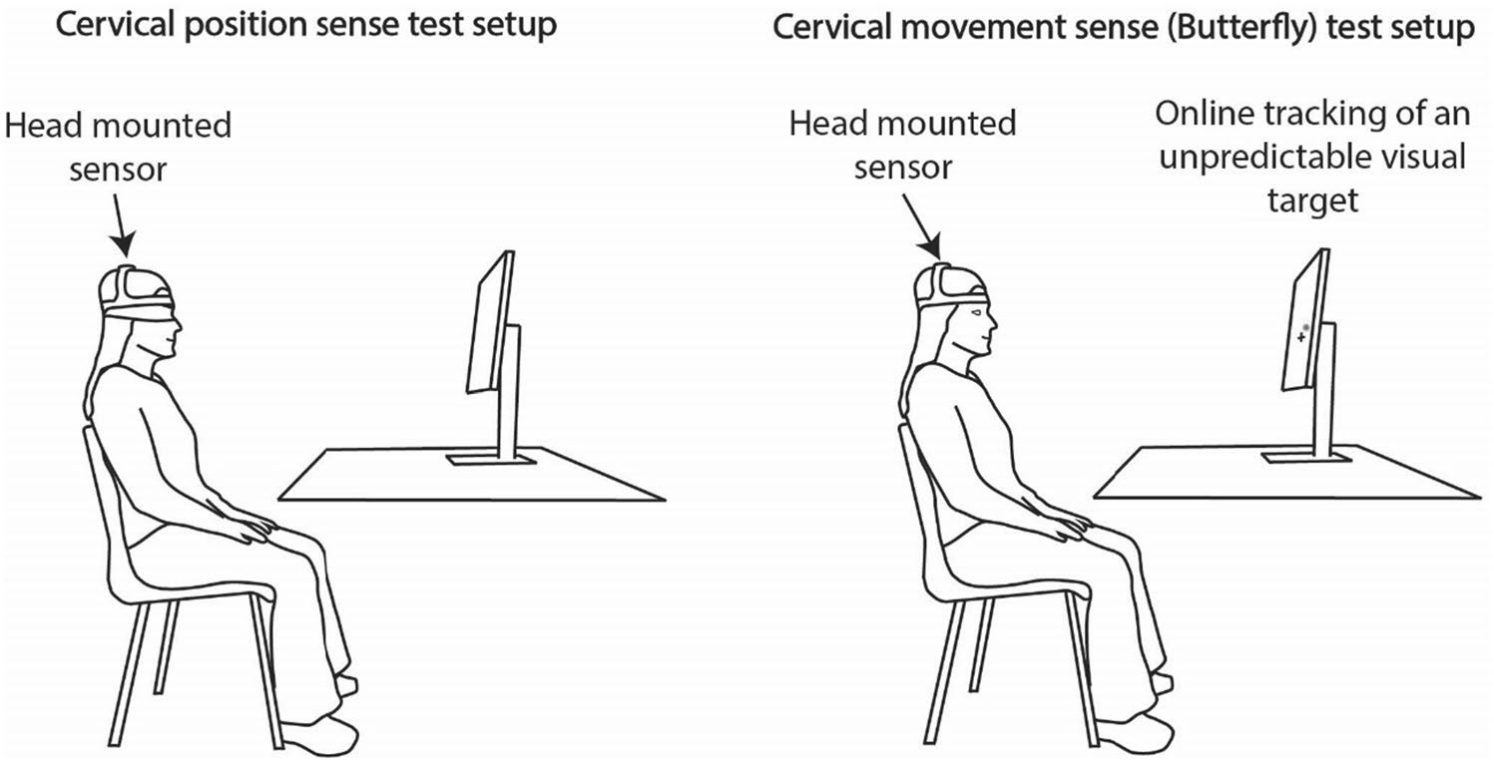
measurement setup.

Patients with neck pain performed cervical position sense test (head-to-neutral relocation test)^50^ and cervical movement sense test (The Butterfly test)^14^ in a setup presented in Fig. 2. Order of performing the two tests was random between patients.

Before performing cervical position sense test, head and neck of each patient were placed to a neutral position serving as a reference. Three repetitions of slow head movements to the end range of motion to both rotations, flexion, or extension and back to neutral position were performed in a random order. All patients were blindfolded during each trial. Head movements were measured by the inertial motion unit (NeckGear, NeckCare ehf, Kopavogur, Iceland) positioned on the patient’s head.

Cervical movement sense test was performed using The Butterfly method described in detail elsewhere^51,52^. During the test, the goal of each patient was to track an unpredictably moving target with their head and neck as accurately as possible. Three different target movement trajectories of increasing difficulty (easy, medium and difficult) were used, each repeated three times. The three difficulty levels differed in predefined velocities at which the target moved through different trajectories. In addition, the difficulty level increased with introducing higher number of movement direction changes. Patients were naïve to the target movement trajectory characteristics. Target movement trajectories and test duration were predefined by the NeckSmart software (NeckSmart, NeckCare ehf., Kopavogur, Iceland).

### Signal analysis

Accuracy of cervical position sense was described using three different parameters expressed in angular degrees (°); mean of the absolute cervical spine relocation deviation from the reference position for three trials for each assessed direction (absolute error), average magnitude of under and overestimation of reference position after cervical spine relocation (constant error) and variability of three consecutive trials expressed as two standard deviations (variable error). All signal analysis and calculations of parameters were performed in NeckSmart software (NeckSmart, NeckCare ehf., Kopavogur, Iceland).

Head and neck movements during the movement sense test were analyzed using the following parameters calculated in NeckSmart software (NeckSmart, NeckCare ehf., Kopavogur, Iceland): average deviation of the head and neck position away from the target during each trial (amplitude accuracy), mean time spent on the target during each trial expressed as percentage of trial duration (time-on-target), time the head and neck spent behind the target expressed as percentage of trial time (undershoot) and in front of the target expressed as percentage of trial time (overshoot), and jerkiness of head and neck movement (smoothness of movement) for each trial were calculated. Averages of three trials for all parameters were used for further analysis.

### Statistical analysis

Differences in VAS score between the enrolled groups were analysed using analysis of variance in a SPSS statistical software (SPSS 23.0 software, SPSS Inc., Chicago, USA). In order to identify latent information provided by the position sense test’s and movement sense test’s parameters, principal component analysis was applied using a SPSS statistical software. As collinearity between parameters was observed, Promax rotation with Kaiser Normalization was used. Only principal components with eigenvalue higher than 1 were used for further analysis. Furthermore, weights of individual parameters were calculated and treated as nonsignificant when they were lower than 0.4. For each individual component the amount of explained variance was calculated. In addition, correlation analysis between individual components was performed using Pearson correlation coefficient (*r*) and treated as no correlation for *r* < 0.3, small correlation for 0.3 < *r* < 0.5, medium correlation for 0.5 < *r* < 0.7 and high correlation for *r* > 0.7^53^.

## Data Availability

The datasets generated during and/or analyzed during the current study are available from the corresponding author on reasonable request.
